# Medication-Related Osteonecrosis of the Jaws: Considerations on a New Antiresorptive Therapy (Denosumab) and Treatment Outcome after a 13-Year Experience

**DOI:** 10.1155/2016/1801676

**Published:** 2016-10-24

**Authors:** Gianfranco Favia, Angela Tempesta, Luisa Limongelli, Vito Crincoli, Eugenio Maiorano

**Affiliations:** ^1^Department of Interdisciplinary Medicine, Complex Operating Unit of Odontostomatology, “Aldo Moro” University, Piazza G. Cesare 11, 70124 Bari, Italy; ^2^Department of Emergency and Organ Transplantation, Operating Unit of Pathological Anatomy, “Aldo Moro” University, Piazza G. Cesare 11, 70124 Bari, Italy

## Abstract

Medication-related osteonecrosis of the jaw (MRONJ) is a serious complication in patients receiving antiresorptive therapies for bone neoplastic localizations and osteoporosis. The aim of this study was to evaluate the clinicopathological features of MRONJ in a cohort of patients treated by new antiresorptive drugs (denosumab) and the corresponding outcome after 13-year maximum follow-up. Overall, 244 patients affected by MRONJ were treated from 2003 to 2015. After clinical and radiological examinations, all lesions were staged according to a dimensional staging system and then surgically treated. All the denosumab-related lesions were classified as stage II or III, thus requiring a more or less invasive surgical approach, despite the results of many recent studies, which suggested a conservative medical approach with early resolution for MRONJ in patients on denosumab. In the current series, 86.9% of treated lesions showed complete clinical and radiological healing, while 13.1% recurred; all recurrences were detected in patients who could not interrupt chemotherapy, steroids, and/or antiresorptive drugs administration due to their general conditions. In conclusion, all oral specialists should be aware of the MRONJ risk among patients taking new antiresorptive drugs; moreover, our protocol based on surgical treatment guided by dimensional staging could be considered effective in view of the low recurrence rate.

## 1. Introduction

Osteonecrosis of the jaw (ONJ) is a well-known complication of antiresorptive or antiangiogenic therapy for the management of osteoporosis and other cancer-related conditions, including hypercalcemia of malignancy and bone metastases of solid tumours such as breast, prostate, and lung cancer, and for the management of lytic lesions in the setting of multiple myeloma [1]. Such lesion was initially termed bisphosphonate-related osteonecrosis of the jaw (BRONJ) as it usually followed the administration of different types of bisphosphonates (BPs). Subsequently, with the advent of new classes of antiresorptive or antiangiogenic medications, such as denosumab, sunitinib, bevacizumab, and ipilimumab, giving rise to similar complications [2–4], the American Association of Oral and Maxillofacial Surgeons (AAOMS) in 2014 recommended the change in the nomenclature into medication-related osteonecrosis of the jaw (MRONJ) [5].

Moreover, AAOMS has modified the MRONJ definition to distinguish this condition from other bone anomalies and, accordingly, patients may be considered to harbour MRONJ if all the following characteristics are present: current or previous treatment with antiresorptive or antiangiogenic agents, exposed necrotic bone or bone that can be probed through an intraoral or extraoral fistula in the maxillofacial region that has persisted for longer than 8 weeks, no history of radiation therapy to the jaws, or obvious metastatic localization to the jaws [5].

BPs are nonmetabolized analogues of pyrophosphate, which prevent osteoclast-mediated bone resorption [6]. In the past, they represented the first-line antiresorptive agents for the management of postmenopausal osteoporosis and for the treatment of bone metastases of solid tumours [7]. More recently, additional molecules have been introduced, such as denosumab, a subcutaneously dosed monoclonal antibody against the receptor activator of nuclear factor-*κ*B ligand (RANKL), which inhibits osteoclast functions and associated bone resorption.

Patients on denosumab for metastatic bone disease receive 120 mg subcutaneously every 4 weeks, while 60 mg subcutaneously every 6 months is used for the management of patients with osteoporosis/osteopenia. The half-life of denosumab is 26 days, while the half-life of BPs ranges from 10 to 12 years. Moreover, differently from BPs, denosumab does not seem to accumulate in the bone, thus leading many authors to maintain that denosumab-related MRONJ are less aggressive and require a more conservative therapeutic approach [8–10].

MRONJ often follows oral surgery or traumatic injuries; however, MRONJ was also detected in patients who had not undergone any invasive dental procedures during the treatment with BPs or denosumab [11].

There are still controversies about the treatment of MRONJ with regard to drug discontinuation, medical therapy, surgery, or other therapies [12]. Also, a large variety of treatment modalities have been proposed, including conservative medical management, mainly repeated cycles of antibiotics, distinct types of surgery (surgical debridement or marginal resection), or other noninvasive therapies, such as hyperbaric oxygen, ozone therapy, and low-level laser therapy for biostimulation [13].

The AAOMS has proposed a staging system to select the best treatment strategy [5]: patients with stages I and II MRONJ should be treated using a conservative approach to prevent the progression of the lesions and limit complications related to chronic infection. Such treatments include conservative debridement of bone sequestra, local irrigation with povidone-iodine, daily rinsing with 0.12% chlorhexidine mouthwash, antibiotic therapy, and pain control [5]. In cases of stage III lesions, marginal resection or surgical debridement is always indicated and the goal of surgery should be to eliminate necrotic bone, acting as foreign material and thus increasing the risk of infection [1].

Franco et al. in 2014 [14] proposed a dimensional staging of MRONJ to more appropriately assess the therapeutic strategy (Table 1).

The aim of this study was to report all cases of MRONJ, treated from 2003 to 2015, in the Odontostomatology Unit of the University of Bari, with specific attention to those occurring in denosumab-treated patients, and to highlight the results of our treatment strategies after a 13-year experience.

## 2. Materials and Methods

Patients with a diagnosis of MRONJ, referred to the Odontostomatology Unit of the University of Bari, Italy, from 2003 to 2015, currently or previously on therapy with BPs and/or other antiresorptive drugs for neoplastic diseases or osteoporosis, with presence of oral exposed necrotic bone or bone that can be probed through a fistula that has persisted for longer than 8 weeks, were included in this study; patients who had received radiation therapy in the oral and maxillofacial area were excluded, leading to a 244-case study cohort.

**Figure 1:**
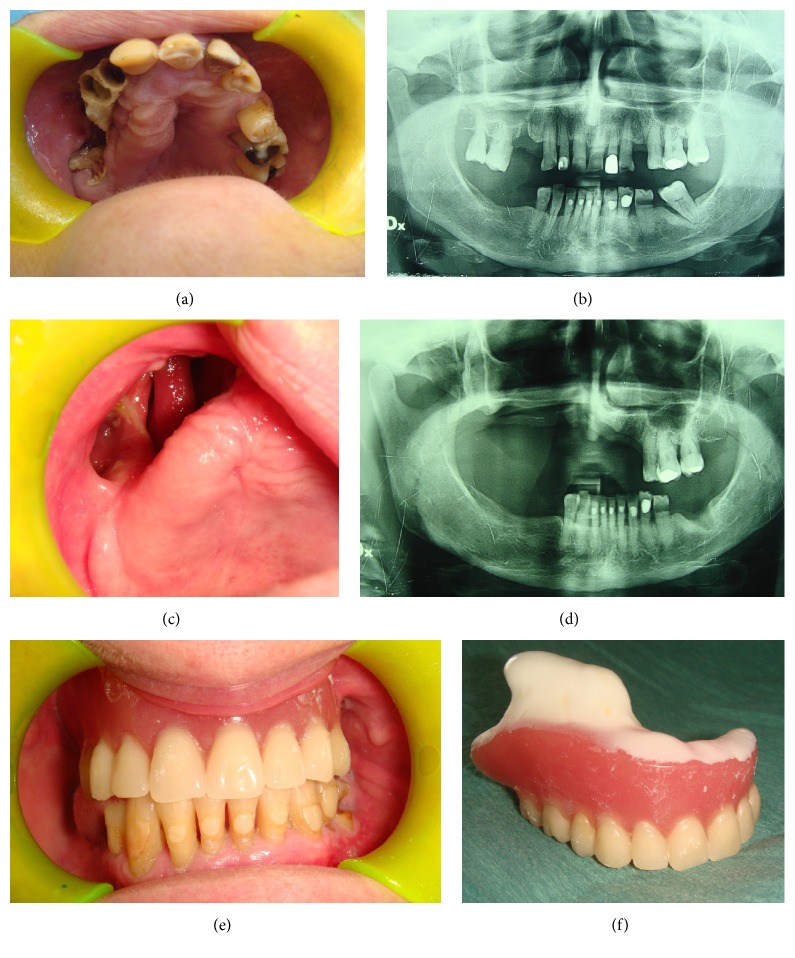
At clinical examination, presence of necrotic bone exposure (a) classified as stage III MRONJ depending on the dimensional staging system on the right maxilla in a female 67-year-old patient affected by breast cancer, who underwent denosumab administration 11 times. Rx OPT shows bone support reduction on the right maxilla. Patient referred the spontaneous loss of 4 teeth (1.7, 1.6, 1.3, and 1.2) after she underwent the orthopantomography, probably due to the progressive bone necrosis and resorption (b). After 16-month follow-up, clinical (c) and radiological (d) healing of the patient who underwent a removable prosthetic restoration (e-f).

**Figure 2:**
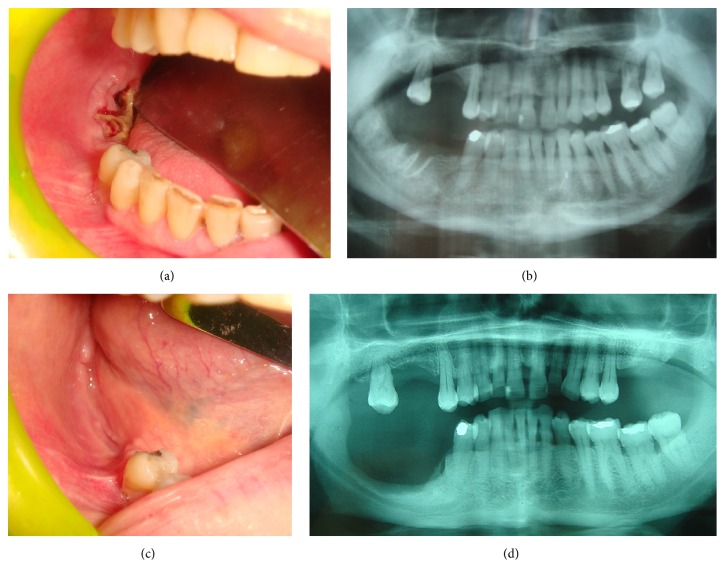
At clinical examination, presence of postextractive necrotic bone exposure on the right mandible (a) of a female 51-year-old patient affected by breast cancer who underwent denosumab administration 9 times. Rx OPT confirms the presence of an area of bone alteration (b) which was diagnosed as stage III MRONJ accordingly with the dimensional staging systems and was surgically treated. After 12-month follow-up, clinical (c) and radiological (d) healing of the treated lesion without signs of recurrence is evident.

A detailed medical history was recorded for each patient, with specific regard to the primary disease and related therapies (BPs, antiresorptive and/or antiangiogenic drugs), dose and duration of treatment, other comorbidities, and related drugs administration. Subsequently, signs of bone necrosis and clinical symptoms were evaluated, together with the site and size of the lesions and possible triggering events. After radiographic evaluation, including orthopantomography and Enhanced Multislices Spiral Computed Tomography with 3D reconstruction, all lesions were staged (Figures 1 and 2) according to both the AAOMS [5] criteria and the dimensional staging [14], the latter being used for the subsequent choice of the surgical approach. Upon consultation with the treating physician, patients suspended any BPs/antiresorptive/antiangiogenic administration not less than 3 months before surgical treatment; chemotherapy and corticosteroids administration were suspended 3–5 days before the surgical procedure and until wound healing, to reduce the recurrence risk. Patients were also administered at least 3 cycles of antibiotic therapy before treatment, consisting in a combination of ceftriaxone (1 g/i.m. daily) and metronidazole (500 mg/per os twice a day) for 8 days with 10 days of interruption after each cycle. The surgical treatment was based on the dimensional staging: surgical debridement for stage I lesions, small open-access surgery with piezosurgery of bone margins for stage II, and wide open-access surgery with bone extensive resection (Figure 2) and piezosurgery of bone margins for stage III lesions [14]; a gel compound with hyaluronic acid and aminoacids was put into the residual bone cavity and on the suture stitches to reduce the wound healing time. All surgical specimens were then sent for histopathological examination.

Each patient underwent an accurate clinical follow-up on a weekly basis in the first month and then a clinicoradiological follow-up at 1, 3, 6, and 12 months after surgery (Figures 1 and 2). If deemed necessary, patients restarted BPs/antiresorptive/antiangiogenic treatments not less than 1 month after surgery.

After one-year follow-up, lesions were considered successfully treated if they completely healed based on clinical or radiological features, or if the lesions could be downstaged according to the AAOMS criteria (Figures 1 and 2); MRONJ occurring at the same site during the clinicoradiological follow-up were considered “recurrences.”

This study was performed in accordance with the principles of the Declaration of Helsinki and has been approved by our institution's ethical committee (study number 4599, Prot. 1528/C.E.); the patients released informed consent on diagnostic and therapeutic procedures and possible use of the biologic samples for research purposes.

## 3. Results

Overall, 244 patients affected by MRONJ (184 females, 60 males) were treated at the Odontostomatology Unit of the University of Bari from 2003 to 2015. Among them, 72 (29.5%) were osteoporotic patients, while 172 (70.5%) were oncologic patients suffering from different malignancies: 75 patients were affected by breast cancer, 53 by multiple myeloma, 23 by prostatic cancer, 6 by lung cancer, 6 by leukaemia/lymphoma, 5 by renal cancer, and 4 by thyroid cancer. Patients had been given different types of antiresorptive drugs, zoledronate being the most frequently used, followed by alendronate, denosumab, clodronate, risedronate, ibandronate, and pamidronate, as illustrated in Table 2.

Moreover, parenteral administration of antiresorptive drugs was referred by 187 (76.6%) patients and oral administration by 57 (23.4%). Also, 54 patients (45 oncologic, 9 osteoporotic) presented multiple MRONJ lesions and, therefore, a total of 322 lesions were treated, 207 in the mandible and 115 in the upper jaw. Tables 3 and 4 illustrate the correlations between the staging of the lesions (according to both the AAOMS criteria [5] and the dimensional staging system [14]) and the different antiresorptive agents used.

**Figure 3:**
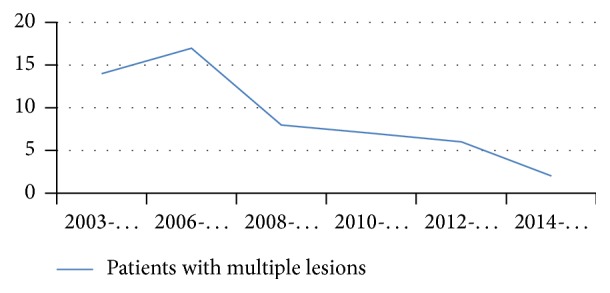
Progressively decreasing number of patients with multiple MRONJ lesions from 2003 to 2015.

We also assessed the distribution of the 45 patients affected by multiple MRONJ according to the year of presentation and the results are synthetically reported in Figure 3.

**Figure 4:**
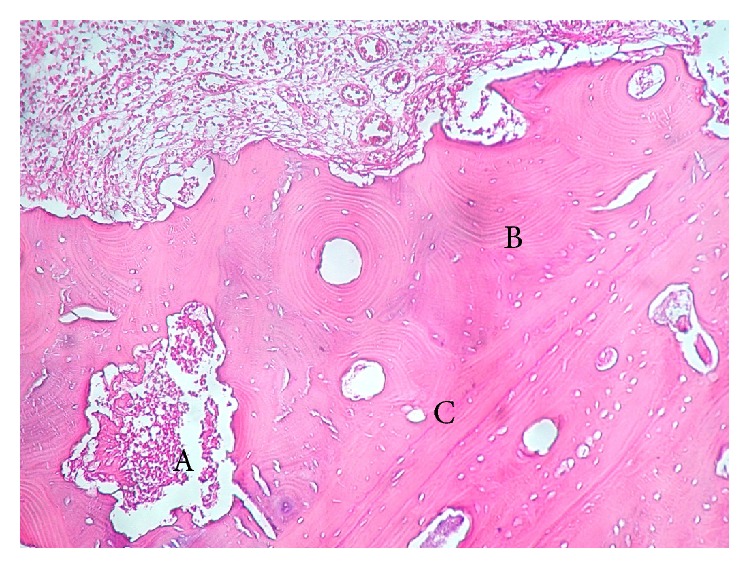
Histopathological features of MRONJ in a patient treated by denosumab. Abundant inflammatory cell infiltration and basophilic bacterial colonies (A) interspersed with necrotic debris are evident. Also, nonnecrotic tissues containing larger amounts of lamellar bone (B), fewer and smaller Haversian canals (C), and larger and thicker osteons are detectable. Haematoxylin-Eosin, 10x.

The histopathological analysis of the surgical samples highlighted typical features of MRONJ, as already reported [15]: extensive bone necrosis, without residual osteocytes/osteoblasts, large and empty Haversian canals, abundant inflammatory cell infiltration, and several basophilic bacterial colonies interspersed with necrotic debris. Also, nonnecrotic tissues containing larger amounts of bone, fewer and smaller Haversian canals, larger and thicker osteons, osteocytes of larger diameter, relative paucity of osteoclast-like cells of relatively smaller size, and reduction of the marrow spaces were detected (Figure 4). Histopathological examination demonstrated complete overlap of the morphological alterations occurring in the lesions of patients treated by denosumab, in comparison with those treated by BPs.

During follow-up (mean: 16 months, range: 10–37) 280 treated lesions (86.9%) showed complete clinical and radiological healing, while 35 lesions (13.1%) recurred. Among the recurrences, 1 case was detected in a patient on denosumab (6% of the denosumab lesions) and the remaining were in patients on BPs (15% of the BPs lesions); 6 patients died of complications related to their oncologic disease. All recurrences were observed in patients who could not interrupt chemotherapy, corticosteroids, and/or antiresorptive drugs administration due to their general conditions.

## 4. Discussion

Antiresorptive agents that target osteoclasts, thereby inhibiting bone resorption and subsequent bone loss, currently are considered the cornerstone for the treatment and prevention of bone metastases of solid tumours and osteoporosis [16, 17]. In the past, intravenous and oral BPs were considered the gold standard for the treatment of such conditions; more recently, other molecules have been proposed, such as the fully human monoclonal antibody denosumab, which was approved by the FDA in 2010 for the treatment and prevention of osteoporosis and bone metastases in oncologic patients.

Although BPs are nonmetabolized analogues of pyrophosphate, while denosumab is a monoclonal antibody against the receptor activator of nuclear factor-*κ*B ligand (RANKL), both share the same mechanism of action, consisting in the inhibition of osteoclast functions and associated bone resorption.

Several clinical trials showed that denosumab is more effective than BPs for the prevention of skeletal-related events in patients with metastatic bone disease and in reducing the risk of bone fractures in osteoporotic patients [18–22]. Moreover, denosumab has a half-life of several weeks and is not eliminated via the kidneys, differently from BPs, which are primarily excreted renally, bound to hydroxyapatite, and may remain sequestered in bones for many years [23]; consequently, the effects of denosumab take place more quickly and may be more rapidly reverted following drug-suspension, thus making this drug a better therapeutic option, especially for patients more prone to develop BPs-related complications, such as MRONJ, or those with compromised renal function.

Cases of denosumab-related MRONJ have been reported during randomized clinical trials for the treatment of patients with metastatic bone disease [18–20, 24–28], the majority of them being associated with known risk factors, consisting in invasive dental procedures and poor oral hygiene [24], and the duration of treatment [29].

The current study is based upon the analysis of 244 patients affected by 322 MRONJ lesions, treated from 2003 to 2015, who had undergone different antiresorptive drugs administration, either parenterally (76.6%) or per os (23.4%), with zoledronate being the most frequently administered (63.1%), especially in oncologic patients.

Tables 3 and 4 show the distribution of the studied cases according to the type of antiresorptive drug and the severity of lesions staged according to AAOMS [5] and to Franco et al. [14]. Although statistical analyses were unsuitable, due to the limited number of patients treated by denosumab, some preliminary considerations may be drawn. In our experience all the denosumab-related lesions were classified as stage II (12.5%) or III (87.5%) with either staging system, thus requiring surgical treatments, which were more invasive for stage III lesions. Furthermore, while 34 recurrences were detected in 228 patients on BPs (14.95% recurrence rate), such complication occurred only in 1 patient of 16 on denosumab (6.25 recurrence rate).

Such data, though requiring powerful statistical confirmation on larger case series, seem to indicate that MRONJ lesions arising in patients on denosumab are more frequently detected in stages II and III, according to both staging systems. Furthermore, we have no knowledge of previous reports dealing with the recurrence rates of treated MRONJ lesions in patients on either BPs or denosumab; nevertheless, the results of the current study indicate that such adverse event is quite rare (15%) in patients on BPs and even rarer (6%) in those on denosumab. Such low recurrence rates also support the effectiveness of the staging system proposed by our group [14] for the selection of the most appropriate treatment based on the extension of the lesions.

Although the first MRONJ case was reported over a decade ago, the pathophysiology of this disease has not been fully elucidated. Altered bone remodeling, osteoclastic or angiogenesis inhibition [5], and relative reduction of blood vessels in MRONJ patients have been reported as possible pathogenetic factors [30]. Favia et al. suggested that reduced angiogenesis following BPs therapy is not mandatory for ischemic-necrotic changes to occur, the latter possibly taking place as a consequence of the expansion of the nonosteonic bone compartment, due to the reduced osteoclastic effects without concurrent and adequate increased blood supply [15]. In the current study we were able to detect similar morphological changes in the tissues from the lesions of both patients with BPs-related and denosumab-related MRONJ; on these premises, we may hypothesize that the above mechanism may be pathogenetically involved in the origin of denosumab-driven osteonecrosis of the jaws.

Recent studies comparing the incidence of MRONJ in cancer patients treated by denosumab or zoledronate showed a slightly increased incidence in the former group (1.7–1.8% versus 1.3%) [24, 31]. Moreover, the risk of developing MRONJ also was significantly increased among patients who received any antiresorptive therapy for metastatic bone disease, in comparison with patients affected by osteoporosis/osteopenia [32], thus suggesting that concomitant or additional treatments, such as chemotherapy, or increased doses of antiresorptive agents may be responsible for such increased risk. Though lacking statistical confirmation, the results of this study provide additional evidence to such hypothesis and also show that the same may also apply to patients undergoing denosumab therapy.

It is known that denosumab has a shorter (26-day) half-life in comparison with BPs (10–12 years) and that it does not accumulate into bone and becomes inert within 6 months after the last administration. Several studies showed that patients on denosumab develop less severe MRONJ lesions than those on BPS and that drug discontinuation, either before an invasive dental procedure or after their development, is effective in preventing such lesions [5, 33] and may show faster resolution of MRONJ, compared to patients on zoledronate [24]. Also, Stopeck et al. maintained that MRONJ occurring in patients on denosumab more frequently were mild or moderate in severity and could be treated conservatively by mouth rinses and antibiotics or, occasionally, by limited surgical treatments (i.e., sequestrectomy, debridement, and curettage) [18], while other studies reported spontaneous resolution of MRONJ 7, 11, 15, and 18 months after the discontinuation of denosumab [25, 26, 34].

The results of the current study are not in complete agreement with the above reported data: no patient on denosumab was found in stage 0 or I, all of them were treated by more or less invasive surgery, and some of them also developed MRONJ recurrence after treatment, though at lower rates than patients on BPs. More importantly, the lack of relevant morphological differences of MRONJ lesions occurring in patients on denosumab, in comparison with those on BPs, apparently gives support to a common pathogenetic mechanism responsible for osteonecrosis and subsequent superinfection. If these data will receive further support, denosumab-related MRONJ will deserve the same attention as that reserved to BPs-treated patients, at least in terms of clinical management and surgical treatment of larger lesions, staged according to the model we proposed. Also, prevention or early identification of MRONJ in patients being treated by denosumab should rely upon the same tools adopted for those treated by BPs, including patients' and practitioners' consciousness of denosumab-related adverse events and the efficacy of dental hygiene and dietary supplements (e.g., calcium and vitamin D) for their prevention. Also, recurrent denosumab-related MRONJ only occurred in cancer patients and, more frequently, in those who could not discontinue concomitant treatments (antineoplastic, steroids, etc.), pointing at the need of prolonged follow-up for these patients.

Finally, we have showed (Figure 3) consistently decreased occurrence of multiple MRONJ lesion over the last 12 years; this favorable trend may be attributable to the positive effects of prevention and should encourage further dissemination of data on MRONJ among general practitioners and general dentists.

## 5. Conclusions

Since the first reports in 2003, thousands of cases of MRONJ have been published in the English literature, but the pathogenesis of MRONJ, its treatment options, and patients' outcome still remain complex and multifactorial and the continuous introduction of new antiresorptive drugs, such as denosumab, makes the situation even worse. In our experience, patients treated with denosumab presented generally with stages II-III lesions, thus requiring surgical treatments and not only conservative management, as reported in many previous studies. In view of the limitations of this study, mainly due to the restricted number of patients on denosumab who developed MRONJ, attentive description of additional series of cases is required to possibly confirm our preliminary findings. Such new studies should possibly confirm the effective role of prevention to at least detect lesion at a smaller size and reduce recurrence rates and of the proposed staging system to drive the choice between surgical versus nonsurgical treatment options.

## Figures and Tables

**Table 1:** Dimensional staging of MRONJ and corresponding treatment options, as proposed by Franco et al. [14].

	Clinical and radiological findings of MRONJ	Treatment
*Stage 0*	No bone exposure with nonspecific radiographic findings, such as osteosclerosis and periosteal hyperplasia, and nonspecific symptoms, such as pain	Medical therapy and clinical-radiological follow-up
*Stage I*	Bone exposure and/or radiographic evidence of necrotic bone, or persisting alveolar sockets < 2 cm in the major diameter, with or without pain	Medical therapy, surgical debridement, and low-level laser therapy (LLLT)
*Stage II*	Bone exposure and/or radiographic evidence of necrotic bone between 2 and 4 cm in maximum diameter, with pain responsive to NSAIDs and possible abscesses	Medical therapy and small open-access surgery with piezosurgery of bone margins
*Stage III*	Bone exposure and/or radiographic evidence of necrotic bone > 4 cm in the maximum diameter, with strong pain, responsive or not to NSAIDs, abscesses, orocutaneous fistulas, and/or maxillary sinus and mandibular nerve involvement	Medical therapy and wide open-access surgery, with extensive maxillary (Caldwell-Luc technique) or mandibular resection, and piezosurgery of bone margins

**Table 2:** Patients' antiresorptive therapy.

Drug prescribed	Oncologic patients		Osteoporotic patients		Total	
	(172)		(72)		(244)	
	*N*	%	*N*	%	*N*	%
Zoledronate	149	86.6%	5	6.9%	154	63.1%
Denosumab	11	6.5%	2	2.9%	13	5.4%
Denosumab + zoledronate	3	1.7%	—	—	3	1.2%
Alendronate	—	—	34	47.2%	34	13.9%
Clodronate	4	2.3%	10	13.9%	14	5.7%
Risedronate	3	1.7%	7	9.7%	10	4.1%
Ibandronate	1	0.6%	7	9.7%	8	3.3%
Pamidronate	1	0.6%	—	—	1	0.4%
Off-label therapy	—	—	7	9.7%	7	2.9%

**Table 3:** MRONJ staging in oncologic patients.

Antiresorptive drug	AAOMS staging system [5]				Dimensional staging system [14]			
	Stage 0	Stage I	Stage II	Stage III	Stage 0	Stage I	Stage II	Stage III
Zoledronate	1	13	126	76	1	22	66	127
Denosumab			4	7			3	8
Zoledronate + denosumab			2	1			1	2
Clodronate			4			2	1	1
Risedronate		2	1			1		2
Ibandronate			1				1	
Pamidronate		1					1	

**Table 4:** MRONJ staging in osteoporotic patients.

Antiresorptive drug	AAOMS staging system [5]				Dimensional staging system [14]			
	Stage 0	Stage I	Stage II	Stage III	Stage 0	Stage I	Stage II	Stage III
Zoledronate		3	2			4	1	
Denosumab				2				2
Alendronate		3	31	5		10	15	14
Clodronate			9	2		3	4	4
Risedronate		1	6	4		1	5	5
Ibandronate			7	1			5	3
Off-label			4	3			5	2
